# Effect of Acrylamide and Mycotoxins in SH-SY5Y Cells: A Review

**DOI:** 10.3390/toxins16020087

**Published:** 2024-02-06

**Authors:** Luna Bridgeman, Cristina Juan, Houda Berrada, Ana Juan-García

**Affiliations:** Laboratory of Food Chemistry and Toxicology, Faculty of Pharmacy and Food Science, University of Valencia, Av. Vicent Andrés Estellés s/n, Burjassot, 46100 València, Spain; luna.birdgeman@uv.es (L.B.); cristina.juan@uv.es (C.J.); houda.berrada@uv.es (H.B.)

**Keywords:** acrylamide, mycotoxins, food processed contaminants, neurotoxicity, in vitro

## Abstract

Thermal processes induce the formation of undesired toxic components, such as acrylamide (AA), which has been shown to induce brain toxicity in humans and classified as Group 2A by the International Agency of Research in Cancer (IARC), as well as some mycotoxins. AA and mycotoxins’ toxicity is studied in several in vitro models, including the neuroblastoma cell line model SH-SY5Y cells. Both AA and mycotoxins occur together in the same food matrix cereal base (bread, pasta, potatoes, coffee roasting, etc.). Therefore, the goal of this review is to deepen the knowledge about the neurological effects that AA and mycotoxins can induce on the in vitro model SH-SY5Y and its mechanism of action (MoA) focusing on the experimental assays reported in publications of the last 10 years. The analysis of the latest publications shows that most of them are focused on cytotoxicity, apoptosis, and alteration in protein expression, while others are interested in oxidative stress, axonopathy, and the disruption of neurite outgrowth. While both AA and mycotoxins have been studied in SH-SY5Y cells separately, the mixture of them is starting to draw the interest of the scientific community. This highlights a new and interesting field to explore due to the findings reported in several publications that can be compared and the implications in human health that both could cause. In relation to the assays used, the most employed were the MTT, axonopathy, and qPCR assays. The concentration dose range studied was 0.1–10 mM for AA and 2 fM to 200 µM depending on the toxicity and time of exposure for mycotoxins. A healthy and varied diet allows the incorporation of a large family of bioactive compounds that can mitigate the toxic effects associated with contaminants present in food. Although this has been reported in some publications for mycotoxins, there is still a big gap for AA which evidences that more investigations are needed to better explore the risks for human health when exposed to AA and mycotoxins.

## 1. Introduction

**Figure 1:**
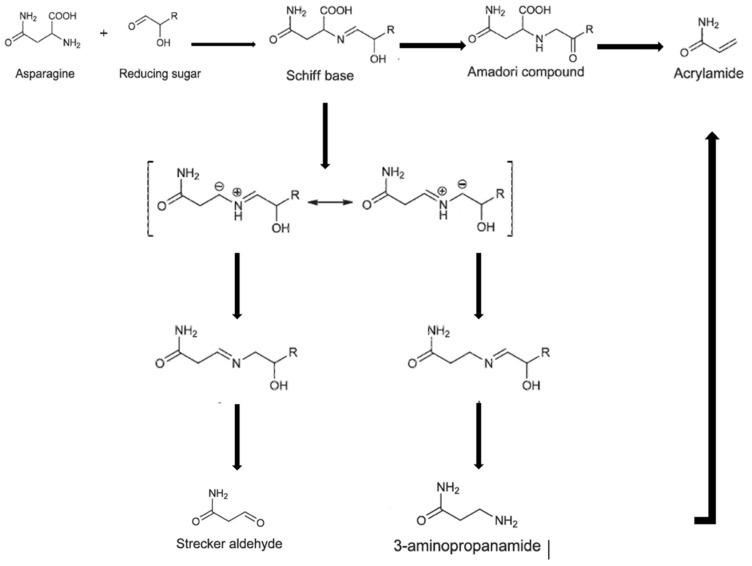
Proposed mechanism for AA formation as a side reaction of the Maillard reaction.

The food baking process is the most important sub-process responsible for the main chemical, physical, and sensory properties of the final product, as well as the development of bioactive and antioxidant compounds [1,2,3]. However, this thermal process induces the formation of unwanted toxic components, including acrylamide (AA), due to the Maillard reaction occurring at high temperatures [1,4,5,6]. In detail, the Maillard reaction involves three major steps: (i) condensation of free amino groups (such as asparagine) with reducing sugars (glucose and fructose) to form acrolein; (ii) Strecker degradation of amino acids to aldehydes and ammonia; and (iii) brown nitrogenous compounds combining with acrylic acid to form AA (Figure 1) [7]. 

The compound AA is a colorless and odorless, highly reactive, and water-soluble crystalline compound with a low molecular weight of 71.08 kDa. AA is used in many different industrial areas, such as water treatment and the production of paper, fabrics, or cosmetics, and is widely employed in laboratories for gel chromatography [8]. The widespread use of AA causes a high level of occupational exposure by inhalation and skin contact [9]. Furthermore, in 2002, Swedish researchers reported that several heat-treated carbohydrate-rich foods contained significantly higher levels of AA than other known food carcinogens [6]. However, several factors can influence the level of AA formation in foods, such as the type of raw materials, product composition, pH, and moisture. In addition, the highest levels of AA are detected in foods derived from thermal processes involving the frying and browning of potatoes, cocoa beans, and coffee roasting, and the baking of cereals and cakes [10]. It is interesting to highlight that AA was classified as a compound “possibly carcinogenic to humans” by the International Agency for Research on Cancer [11] and classified in Group 2A. Likewise, the European Commission (2017) [12] classifies AA as a Class 1B carcinogen and mutagenic substance and Class 2 for reproductive toxicity, while the European Chemicals Agency (2022) [13] has included AA in the list of substances of very high concern. Therefore, it is of great interest, and further studies of AA toxicity are necessarily required to assess the potential risk for human health.

The toxicodynamic characteristics associated with AA are based on rapid absorption in the blood and wide distribution to tissues due to its low molecular weight and high solubility properties [14]. AA is mainly metabolized in the liver and brain by the glutathione-S-transferase enzyme, forming N-acetyl-S-(3-amino-3-oxypropyl)-cysteine [15]. Moreover, AA can be converted by cytochrome P450 2E1 (CYP2E1) to glycidamide (GA), a more reactive epoxide that can react with hemoglobin and DNA [16,17]. Thus, GA is responsible for the mutagenic and carcinogenic effects of AA in vivo [18]. AA has been shown to induce systemic side effects in animals, while brain toxicity has been detected in humans [19,20]. It has also been reported that AA neurotoxicity is associated with the imbalance between oxidation and antioxidant function brought on by lipid peroxidation and a rise in intracellular reactive oxygen species (ROS) [8].

Mycotoxins are toxic compounds produced by various *Aspergillus*, *Penicillium*, *Fusarium*, and *Trichoderma* fungal species that are health-hazardous poisons frequently present in various agricultural products, including grains, nuts, and spices [21]. These toxins pose significant health risks to humans and animals when consumed [22]. Understanding the effects of mycotoxins on different cell types is crucial for assessing their toxicological impact [23,24,25].

Studying mycotoxins in vitro using SH-SY5Y cells allows researchers to assess their potential neurotoxic effects and elucidate the underlying mechanisms of toxicity. These studies involve exposing the cells to various concentrations of specific mycotoxins, such as ochratoxin A (OTA) or fumonisins, and evaluating their impact on cell viability, morphology, proliferation, apoptosis, oxidative stress, neurotransmitter function, and other relevant endpoints [26,27].

Likewise, the European Commission has reported more than 400 alerts for fungal contamination in food and feed, with OTA being one of the most reported toxins, accounting for 10% of the notifications [28].

Nowadays, there is a strong effort toward studying the combination of compounds that can occur in the same food matrix, environment, or are daily ingested through the diet, especially regarding the effects that mixed compounds can cause. AA and mycotoxins neurotoxicity is reviewed in several in vitro models, including undifferentiated and differentiated human neuroblastoma cell line (SH-SY5Y) models. SH-SY5Y cells are used in toxicological research to assess neuronal differentiation, metabolism, and function, as well as in neuroadaptive, neurodegenerative, and neuroprotection processes [29]. The human SH-SY5Y cells are a subclone of SK-NSH, which, once stimulated with retinoic acid (RA), differentiates into dopaminergic neuron-like cells, acquiring many biochemical and functional properties of neurons [30,31].

Moreover, despite numerous reviews offering scientific insights into the chronic exposure [32], toxicity [33,34], presence in food [35], and various extraction procedures [36,37] related to AA and mycotoxins individually, there remains an unaddressed gap in the literature: a review uniting the toxic effects of AA and mycotoxins within an in vitro model, such as SH-SY5Y cells. In addition, a new study establishes the combined effects of these compounds in SH-SY5Y cells, emphasizing the importance of collecting data on AA and mycotoxins individually to understand their mechanisms of action [38]. Furthermore, the interest in the shared toxic effects documented in both acrylamide (AA) and multiple mycotoxins is not just due to their occurrence in our diets but also attributable to the potential combination of these effects, raising important implications for human health, particularly concerning neuronal impacts. Therefore, this review aimed to evaluate the effects of AA and mycotoxins as well as their mechanism of action (MoA) in the human neuroblastoma cell line SH-SY5Y, as reported in studies of the last 10 years.

The results have been organized according to findings of AA and mycotoxins that might coexist together in food and cereal food-based products. Thus, the section has been divided into the following sub-sections: cytotoxicity, apoptosis, oxidative stress, network degeneration, signaling pathways, protein expression, and natural compounds against the adverse effects of AA. 

## 2. Cytotoxicity

### 2.1. Acrylamide (AA)

Non-viable or dead cells are associated with a loss of membrane integrity, which is accomplished by alterations in the movement of molecules either into or out of cells across membranes that have become leaky [39]. Cytotoxicity assays are convenient for understanding the dose- and time-dependent toxicity of chemicals and their reversibility and impacts on the cell cycle. Multiple methods are known, depending on their endpoints; some are based on a visual morphological scoring process (e.g., elution test, direct and indirect contact), while other methods use colorimetric and numbering rapid analysis techniques [i.e., trypan blue exclusion, propidium iodide uptake, 3-(4,5-dimethylthiazol-2-yl)-2,5-diphenyl-2H-tetrazolium bromide (MTT), etc.] [40]. 

In this sense, to find out the cytotoxicity of AA at the neuronal level, several authors investigated the cytotoxicity by MTT assay in SH-SY5Y cells when incubated with different concentrations of AA for 24 h [41]. Reductions in cell viability were detected from 15% to 40% in SH-SY5Y cells treated with AA (from 2 to 5 mM) in a dose-dependent manner [41,42,43]. Furthermore, changes in the shape of cells were detected after exposure to 2.5 or 5 mM of AA, for 24 h, as most cells shrank, and the cell body became round [41]. In the same line of research but one step forwards, the same group of investigators studied the protective effect of curcumin on AA-induced cytotoxicity for which SH-SY5Y cells AA-treated from 2.5 to 10 mM obtained a decrease in the number of living cells from 14% to 61% with respect to the control by a CCK8 assay [42], while the treatment of SH-SY5Y cells with 100 μM of α-lipoic acid (LA) suppressed AA apoptosis induction and the loss of cell viability. 

Similarly, comparing the cytotoxicity of SH-SY5Y and a human glioblastoma cell line (U-1240 MG), in different time- and dose-dependent assays, it was shown that upon treatment with AA from 0.1 to 2 mM up to 72 h, SH-SY5Y viability was significantly reduced up to 40% upon exposure to 2 mM AA; while for U-1240 MG cells, viabilities were significantly reduced up to 92% for that same concentration of AA (2 mM) [44]. Coinciding with the results for SH-SY5Y cells in reducing viability, significance was also reported at 100 µM and after 72 h of exposure [45]. 

In addition to the common cytotoxic assay of MTT, viability alterations produced by AA have been carried out by SF assay [46], and trypan blue and lactate dehydrogenase leakage (LDH) [47]. The effects reported for SH-SY5Y cells by SF assay revealed that viability was altered at high concentrations of AA (10 mM) causing definite cellular damage and cell death at short times of exposure (8 h) [46]; while cytoxicity evaluation by trypan blue exclusion and LDH leakage was reported at the same doses of AA (10 mM) but, in this case, at 6 h with a viability decreased by 38% [47]. Compared with U-1240 MG cells, at 24 h, a reduction in viability of 20% and 35% at 5 and 10 mM was shown, respectively, concluding that the cell viability reduction in U-1240 MG cells was less than that reported for SH-SY5Y cells [47].

Another study carried by Okuno et al. (2006) [48], also in SH-SY5Y cells, showed that AA cytotoxicity was dose (0.5–5 mM) and time (1–24 h) dependent, by the fact that trypan blue exclusion decreased and LDH leakage increased [48]. The WST-8 assay is another assay for cell viability and it was compared with LDH leakage on SH-SY5Y exposed to a concentration range of 1–5 mM AA [49]; the results revealed a WST-8 decrease and LDH increase according to the AA dose, but it helped to confirm the previous results [49]. 

On the other hand, the possibility of SH-SY5Y cells to be differentiated as neurons allowed researchers to study the effect of AA by the LDH assay, revealing that it was largely non-cytotoxic at 1 h of exposure, except at higher doses (10 mM); this was similar to the results when undifferentiated SH-SY5Y cells were tested [50].

Furthermore, Frimat et al. (2010) [51] demonstrated through the viability assay with CellTiter-Blue that an AA concentration of 5 mM reached a 50% inhibition concentration (IC_50_) for SH-SY5Y cells, while 0.26 mM caused a 20% reduction in the network formation equivalent to the control at 24 h of exposure.

In another study, the basal cytotoxicity was determined by measuring the total cellular protein content, and the total protein from the 50 S subunit (TP_50_) and TP_20_ values (1.34 mmol/L and 0.61 mmol/L, respectively) reflected severe and moderate cytotoxicity, respectively, in SH-SY5Y cells exposed to AA [52].

However, other authors showed that 0.5–2.0 mM of AA did not produce any cytotoxicity (assessed by fluorescence microscopy) at 24 h, but concentrations higher than 4 mM caused a significant loss of HuD-positive neurons (58% of control) and a decrease in axon number by 21% with respect to the control [53]. Similarly, as proved by Calcein-AM assay, (a fluorescence-based cell viability assay), 1 mM of AA had no statistically significant toxicity at 48 h in SH-SY5Y cells or the embryonic carcinoma cell line (P19) derived from an embryo-derived teratocarcinoma in mice; however, it was cytotoxic for pheochromocytoma of the rat adrenal medulla (PC12) cells [54]. A study of cytotoxicity through fluorescence microscope observation by cytotoxicity tests using the Live/Dead Cell Staining Kit II following exposure to different concentrations of AA (0.01, 0.28, 7 mM) in the SH-SY5Y cell line at 24 h reported that the ratios significantly decreased at 0.28 mM and 7 mM, respectively [55].

In order to obtain more accurate results, the cytotoxicity of AA on SH-SY5Y cells was studied by MTS assay and the toxicity after 24 h of incubation was evaluated [56]. The amount obtained in the abluminal concentration after 1 h was also measured in the blood–brain barrier (BBB) model, to finally compare the results of cytotoxicity between AA assayed directly in SH-SY5Y after 24 h with the AA obtained after passing through an in vitro BBB (4 d/24 w) model for 1 h. It was proved that AA was cytotoxic at 100 μM in SH-SY5Y at 24 h, but not the abluminal AA concentration after BBB transport [56].

### 2.2. Mycotoxins

#### 2.2.1. Beauvericin (BEA)

Studies on the impact of BEA on SH-SY5Y cells have illustrated varied results, predominantly through the utilization of the MTT assay. Viability was shown to be reduced by 50% during a 72 h exposure at 2.5 µM, with some studies citing a 43% decrease in viability at the highest concentration assayed (2.5 μM) after 48 h of exposure [57,58,59,60]. Diverse findings have been noted regarding IC_50_ values at different exposure times, such as 1.9 µM at 6 h, 1.7 µM at 24 h, and 1.5 µM at 48 h [61,62]. LDH leakage was also found to be induced by 1 µM of BEA, corroborating results obtained by the MTT assay [61].

#### 2.2.2. Deoxynivalenol (DON)

Regarding the effects of DON in SH-SY5Y cells, the MTT assay was carried out for both studies, but with different concentrations and results. The IC_50_ for Pérez-Fuentes N. et al. 2021 [61] was 2.25 µM at 24 h, but 120 µM for Kalagatur et al. [63] at the same time. Pérez-Fuentes N. et al., 2021 [61] did not find any increase in the extracellular LDH level, but Kalagatur et al. [63] reported a directly proportionate extracellular LDH level in comparison to the MTT assay.

#### 2.2.3. Enniatin A and Enniatin B (ENN A and ENN B)

Six hours of ENN A exposure resulted in a 41.0 ± 8.5% drop in cell viability at 2.5 μM and 94.3 ± 1.6% at 5 μM, respectively, with total inhibition at 10 μM. After incubation for 24 and 48 h, cell viability at 2.5 and 5 μM showed a decrease greater than 74.5%, and at the highest tested dose, a complete reduction was observed once more. Concerning ENN B, cell viability did not reach total inhibition at the highest dose at 6 h [61]. Nevertheless, at 24 h, ENN B had an IC_50_ of 0.43 μM. Also, ENN A increased LDH release at 5 and 10 μM at 24 and 48 h. After treatment for 24 h, ENN B increased LDH release by roughly 20% at values higher than 0.25 μM. After 6 h of incubation, at 5 and 10 μM, ENN A showed substantial differences from control cells (79.0 ± 8.9% and 67.7 ± 14.5%, respectively) [61].

#### 2.2.4. Fumonisin B1 (FB1)

Concerning the effects of FB1, several studies investigated the toxic potential of this mycotoxin in SH-SY5Y cells. In total, 100 µM of FB1 decreased the cell viability of SH-SY5Y cells after incubation for 48–144 h [64]. In the same line but at lower exposure times, after 48 h of incubation, FB1 reduced the cell viability at the highest concentration (30 µM), and it appeared to promote cell proliferation at the lowest doses (0.1 µM) after 24 and 48 h. Furthermore, no IC_50_ was found, and in the LDH assay no cytotoxic effects were shown following FB1 treatment [62]. Similarly, in another study, 50 µM treatment of FB1 was compared to the control, and LDH was released at 12 h, but this release decreased at 24 h and at 48 h of treatment and there was no difference from the control [65]. Then, Domijan et al. (2011) [66] investigated how FB1 (at concentrations ranging from 0.5 to 200 M) affected the viability of cell cultures after 24 h of exposure. None of the FB1 concentrations utilized in the experiment caused cell death in neuroblastoma cells. Neuroblastoma cells treated for 24 h with the maximum FB1 dose (200 µM) showed 98.0 ± 1.85% cell survival.

#### 2.2.5. Ochratoxin A (OTA)

Concerning the viability of SH-SY5Y cells treated with OTA, the IC_50_ values observed for 24 h and 48 h were 9.1 μM and 5.8 μM, respectively [67]. A significant decline in viability was noted beyond concentrations exceeding 3.12 μM or 6.25 μM for 24 h and 48 h, resulting in a decrease from 74% to 25% and from 80% to 49% at 24 h and 48 h, respectively [67]. Similarly, less dramatic decreases in mitochondrial activity were observed under different concentrations of OTA treatment (74 ± 12 and 74 ± 44% control at 10 and 100 µM OTA, respectively) at 24 h, and the LDH activities of OTA at 1, 10, or 100 µM were 128 ± 1, 125 ± 2, and 200 ± 1% of the untreated control [68].

#### 2.2.6. T-2 Toxin

Regarding the exposure of SH-SY5Y cells to the T-2 toxin, this resulted in a significant decrease in cell viability, with percentages of 81.9%, 40.8%, and 35.5% observed for cells exposed to 5, 10, and 20 ng/mL of the toxin for 48 h, respectively. Additionally, LDH levels were significantly elevated by 1.8, 2.9, and 3.2 times compared to the control group [69].

#### 2.2.7. Zearalenone (ZEA) and Its Metabolites

Referring to ZEA effects on SH-SY5Y cells, the MTT assay stated that at 6 h of incubation, ZEA did not cause any cell damage. However, it significantly reduced cell viability when the concentration exceeded 20 μM at 24 and 48 h, resulting in a reduction of more than 50% compared to the control cells. The inhibitory concentration (IC_50_) of ZEA was determined to be 17.4 μM [61]. However, after 24 h of ZEA treatment, in a previous study [70] similar results were obtained in SHSY5Y cells with a significant decrease in cell viability even at a lower concentration (25 µM). The viability of SH-SY5Y cells decreased by as much as 86% at 200 µM ZEA [70]. Regarding the cell viability in ZEA metabolites (α-ZEL and β-ZEL), the following IC_50_ values were obtained for α-ZEL: 20.8 at 48 h, 14.0 at 72 h and for β-ZEL: 94.3 at 24 h, 9.1 at 48 h, and 7.5 at 72 h [59].

In summary, we can state that AA produces cytotoxicity in SH-SY5Y cells, at high concentrations (5 mM or higher), and that in lower concentrations it reduces cell viability in a time- and dose-dependent manner (Table 1). On the other hand, mycotoxins produce cytotoxicity at low concentrations of 200 µM or less and this is time- and dose-dependent in some cases. The most cytotoxic compound was ENN B, followed by DON and ENN A. (Table 2). Therefore, AA and mycotoxins directly affect cell viability in all cell models studied; the most sensitive cells exposed to AA were PC12, followed by SH-SY5Y, and U-1240 MG.

## 3. Apoptosis

### 3.1. Acrylamide (AA)

In order to assess cellular apoptosis, the levels of mono- and oligonucleosomes were monitored in SH-SY5Y and U-1240 MG cells treated with AA. This revealed an increase in the enrichment factor of both mono- and oligonucleosomal fragments in both cell lines when exposed to concentrations ranging from 0.5 to 10 mM of AA over a period of 0 to 72 h. In SH-SY5Y cells, the levels of DNA fragments showed a significant increase with longer exposure times and higher concentrations of AA. On the other hand, U-1240 MG cells exhibited apoptotic responses only after 48 h of exposure to all concentrations of AA, except at 0.5 mM [47]. Moreover, when SH-SY5Y cells were treated with 2.5 mM of AA for 24 h, there was an approximately four-fold increase in the total apoptotic rate compared to the control group [38]. However, inhibiting c-Jun N-terminal kinase (JNK) using a nuclear factor κB (NF-κB) inhibitor led to a reduction in both early and late apoptosis in AA-treated cells [41].

To investigate the potential role and underlying mechanism of Sphingosine Kinase 1 (SphK1) in AA-induced nerve injury in SH-SY5Y cells, the expression of SphK1 was examined. It was observed that SphK1 levels decreased in correlation with increasing concentrations of AA. Specifically, after exposure to AA, the apoptosis rates and activation of SphK1 increased proportionally with the AA concentration [72]. At concentrations of 1.25 mM and 2.5 mM, the apoptosis rates were 3.06% ± 0.13% and 6.86% ± 0.67, respectively, while in the SphK1 activator group, the rates at the same concentrations were 2.12% ± 0.33% and 3.53% ± 0.17, respectively [72]. Furthermore, in a related study, Ning et al., 2021 [68], took the investigation a step further by using AA as a model to induce apoptosis in zebrafish, exposing them to 10 mM of AA. This resulted in the observation of AA-induced neuroapoptosis through fluorescent assays in the zebrafish [73].

### 3.2. Mycotoxins

#### 3.2.1. Beauvericin (BEA)

Regarding cell cycle alterations, BEA significantly reduced the percentage of viable cells, producing an increase in apoptotic cell death of 55.9% ± 8.6% [61]. Similar results were obtained by Agahi et al., 2021 [74], who stated that apoptotic/necrotic cells increased for both times of exposure, which was up to 89% for 24 h and up to 38.8% for 48 h. Furthermore, Agahi et al., 2021 [74] observed that after 48 h of exposure, there was a notable increase in necrotic cells at 0.39 and 0.78 μM by almost two times compared to control cells.

#### 3.2.2. Deoxynivalenol (DON)

In the case of DON, Kalagatur et al. [63] reported that a dose of 120 µM induced DNA damage and led to the formation of apoptotic nuclei.

#### 3.2.3. Enniatin A and Enniatin B (ENN A and ENN B)

ENN A and ENN B reduced cell viability, producing an increase in apoptotic cell death of 49.2 ± 8.2% and 46.0 ± 9.3%, respectively [61].

#### 3.2.4. Zearalenone (ZEA) and Its Metabolites

ZEA, on the other hand, did not produce a significant increase in cell death at the concentrations tested [61]. Nevertheless, α-ZEL treated cells increased significantly in apoptotic and apoptotic/necrotic cells at 24 h. And β-ZEL showed a significant increase in apoptotic cells exposed to the highest concentration assayed (12.5 μM) after 24 h and 48 h [74].

AA increases the apoptosis rate in neuronal cells (SH-SY5Y and U-1240 MG cells) proportional to the AA concentration, as well as to the time of exposure. Activating SphK1 could improve the survival rate of SH-SY5Y cells and reduce the apoptotic rate. Also, it has been shown to produce neuroapoptosis in zebrafish (Table 3). Mycotoxins like BEA, ENNs, and ZEA metabolites (α-ZEL and β-ZEL) could reduce the cell viability in SH-SY5Y cells and produce an increase in apoptotic cell death (Table 4).

## 4. Oxidative Stress

### 4.1. Acrylamide (AA)

The study of ROS in the SH-SY5Y cell line revealed an accumulation after AA exposure [46]. These results are supported by those reported indicating an intracellular decrease in glutathione (GSH) production in a dose-dependent manner and increased malondialdehyde (MDA) and ROS generation when SH-SY5Y cells were treated with 2.5 and 5 mM of AA [41]. Furthermore, when SH-SY5Y cells were exposed to AA at 2.5 mM for 24 h, it caused oxidative stress as revealed by the distinct increase in cellular ROS and the MDA level and a significant decrease in GSH content [42].

### 4.2. Mycotoxins

#### 4.2.1. Beauvericin (BEA)

In view of the effects on cytotoxicity and apoptosis that mycotoxins had, oxidative stress was analyzed by several authors. Agahi et al. [75] studied the ROS production and the GSH/GSSG ratio in which BEA produced a slight decrease at 1.25 and 2.5 μM, from 45 to 120 min compared to the control, and GSH/GSSG ratio increased after 24 h in cells exposed to BEA from 103% to 142%. Subsequently, in the same line of investigation, they studied the activity of GPx, GST, CAT, and SOD [76]. The results showed an increase ranging from 9- to 17-fold and from 2- to 9-fold after 24 h and 48 h, respectively. Also, BEA increased GST activity at doses above 0.78 μM for 48 h of exposure by 4–32%. CAT activity was not altered when cells were exposed to the BEA mycotoxin. Finally, SOD activity increased significantly after being exposed to BEA after 48 h by up to 1-fold. In other study [61], the mitochondrial membrane potential measurement was analyzed, concluding that BEA had the capacity to depolarize the mitochondrial membrane at concentrations ranging from 2.5 to 10 μM at 6 h and 24 h.

#### 4.2.2. Deoxynivalenol (DON)

Also, Kalagatur et al. [63] reported that at 120 µM, DON induced ROS and oxidative stress over the induction of LPO and exhaustion of antioxidant enzymes (GSH, CAT, and SOD). Also, DON induced MMP loss [63].

#### 4.2.3. Enniatin B (ENN B)

At 24 h, this emerging toxin affected the ΔΨm value at all of the concentrations tested. ENN B treatment altered ΔΨm at the highest doses employed (5 and 10 μM) after 6 h of incubation and caused differences at 24 h [61].

#### 4.2.4. Fumonisin B1 (FB1)

In terms of oxidative stress, FB1 treatment resulted in a markedly higher production of ROS as compared to control cells. Additionally, they measured the amount of ROS accumulation in the mitochondria and found that FB1 therapy, independent of treatment duration, enhanced ROS accumulation in the mitochondria [65]. It is interesting to note that adding 0.5 µM of FB1 to neuroblastoma cells increased the rate of ROS by 1.2 times. In neuroblastoma cells, increasing the FB1 concentration to 5 and 50 µM did not activate the rate of HEt fluorescence in a dose-dependent manner [66]. On the other hand, at all of the time points (24, 48, 72, 96, 120, and 144 h) and fumonisin concentrations (0.1 to 100 µM) employed, treatment with FB1 had no effect on the formation of ROS [64]. 

When free radical levels are high, ROS can be harmful to cells because they significantly reduce the quantity of endogenous antioxidants. One of the main CNS antioxidant pathways is supplied by GSH. Oxidative stress causes GSH to be oxidized, and CNS disease results from GSH deficiency [67]. Reduced GSH levels (61% of controls) were seen in SH-SY5Y cells following a 144 h incubation period with 100 µM FB1, but not at earlier times or with lower FB1 doses [60]. 

After incubating with 100 µM FB1 for 24 h, SH-SY5Y cells already showed increased MDA concentrations [61]. Following a 72 h incubation period, cells exposed to 10 µM FB1 exhibited elevated MDA levels. MDA levels at the other time periods did not statistically differ from those of controls [61].

In a different study, the neuroblastoma cells’ cellular Ca^2+^ level considerably increased following FB1 exposure in comparison to the control group. Following ER stress, cellular Ca^2+^ is released, which causes the loss of mitochondrial membrane potential and, in neuroblastoma cells, results in cell death [65]. Nevertheless, Pérez-Fuentes N et al., 2021 [61], found that 30 µM FB1 treatment did not result in appreciable alterations in the ΔΨm of SH-SY5Y cells during either of the two incubation periods. While MitoSOX florescence rose at all doses, only the lowest concentration (0.5 µM) had a discernible impact [66].

#### 4.2.5. Ochratoxin A (OTA)

When it came to ROS, 100 µM OTA for 30 and 60 min, followed by loading cells with 2 mM DCF-DA for 30 min, showed that OTA increased the intensity of DCF-DA fluorescence in comparison to the corresponding untreated control cells. This suggests that OTA-treated SH-SY5Y cells may have been exposed to oxidative stress [68].

#### 4.2.6. T-2 Toxin

In this investigation, ROS levels dramatically increased to 3.8 and 5.0 times, respectively, in cells treated with 5 and 10 ng/mL of the T-2 toxin. As a result, for cells treated with 5 and 10 ng/mL of the T-2 toxin, the ratio of GSH to GSSG was drastically reduced to 79.8% and 60.7%, respectively. The ATP content dropped to 66.7% and 51.5% at the 5 and 10 ng/mL concentrations, respectively, whereas the mitochondrial membrane potential decreased to 60.7% and 41.5% at the same concentrations [69].

#### 4.2.7. Zearalenone (ZEA) and Its Metabolites

ZEN administration dramatically boosted the formation of ROS in SH-SY5Y cells in relation to oxidative stress [70]. Additionally, ZEN administration led to a dose-dependent increase in MMP loss and lipid peroxidation [60,61]. Regarding the ZEA metabolites, α-ZEL at 25 μM elevated ROS from 5 to 60 min and moderately decreased from 90 to 120 min in comparison to their control. Conversely, β-ZEL dropped for 12.5 μM over a 90 min period [75]. After 24 h, the GSH/GSSG ratio in cells exposed to mycotoxins in fresh media increased dramatically from 111% to 148%, and for α-ZEL and β-ZEL, from 68% to 131%, respectively [75]. Deepening on our understanding of the harmful consequences of ZEA metabolites, the study conducted by Agahi et al. [76] examined the effects of α-ZEL and β-ZEL on the activity of enzymes in SH-SY5Y cells. The findings showed that α-ZEL and β-ZEL exposure increased the activity of GPx, CAT, and GST at all concentrations examined, and that SOD and α-ZEL increased the activity of SOD and GST after 48 h.

Summarizing, AA produces an increase in cellular ROS and a reduction in intracellular GSH at concentrations higher than 2.5 mM for more than 24 h (Table 5). Mycotoxins (DON, FB1, OTA, T-2 toxin, and ZEA) produced an increase in cellular ROS and in mitochondrial ROS in the case of FB1, and a reduction in the GSH ratio at concentrations of 120 µM for DON, 0.5 µM for FB1, 100 µM for OTA, 5 and 10 ng/mL for T-2 toxin, and 12.5 μM for α-ZEL and β-ZEL (Table 6). 

## 5. Network Degeneration

Another way to assess how AA exerts effects at a neuronal level is by explaining whether it degrades or impedes the development of the neural network during the process of differentiation; a study found that degeneration was not noticeable for the first 48 h after receiving 1 mM AA treatments, but that the network levels had considerably decreased by the 72 h mark [77]. In this instance, network deterioration could not be stopped by co-treatment with BDNF or calpeptin. Similar results were later reported proving that the number of neurites was significantly reduced after 100 nM following 3 days of exposure and after 6 days of exposure to 10 pM of AA [45], showing an impairment in the neurite outgrowth in a time- and dose-dependent manner in future studies [55]. 

Furthermore, 0.5 mM caused a shortening in SH-SY5Y neurite morphology, while 1 and 2 mM of AA resulted in cells with no neurite morphological extensions [44]. Similar results were found when cells were treated with 0.25 mM of AA, causing a 50% neurite degeneration at 24 h [78]. At 96 h with 4 mM of AA, SH-SY5Y neurites were reduced by up to 67.7% [53]. Thus, AA induces network degeneration in a dose- and time- dependent manner [79].

In order to obtain more accurate results, Nordin-Andersson et al., 1998 [79], exposed differentiated human neuroblastoma (SH-SY5Y) cells to a series of contaminants, among them AA, studying general cytotoxicity (IC_20_) and the neurite degenerative effect (ND_20_), concluding after 72 h that the IC_20_ was 670 mM and the ND_20_ 250 mM. Thus, the ND_20_ values for AA were significantly (65%) lower than the IC_20_, concluding the induction of axonopathy. Later on in 2003, during the AA treatment, there were observations of basal cytotoxicity, morphological modifications, and changes in cell physiological and neurochemical activities in differentiated human neuroblastoma (SH-SY5Y) cells [52]. After 72 h of exposure, AA caused a 20% drop in the number of neurites per cell at 0.21–0.25 mM and a 20% decrease in the rate of protein synthesis at 0.17 mmol/L. Moreover, there was a 49% and 38% rise in the baseline intracellular calcium concentration ([Ca^2+^]i) fluxes, measuring 0.25 mmol/L and 0.5 mmol/L, respectively. The SH-SY5Y cells recovered 48 h after the AA exposure was stopped; that is, their basal level of [Ca^2+^], rate of protein synthesis, and number of neurites per cell were all similar to those of control cells.

Taken together, AA impairs neurite outgrowth in the SH-SY5Y cell line during differentiation in a time- and concentration-dependent manner, especially after 72 h of exposure. However, at 48 h after cessation of AA exposure, SH-SY5Y cell recover (Table 7).

## 6. Signaling Pathways

To fully understand how AA interferes at the neuronal level, various studies scrutinized the involvement of different transcription factors crucial in neurodevelopment, cell differentiation, apoptosis, and inflammatory responses. These included cAMP response element-binding (CREB), mitogen-activated protein kinases (MAPKS), JNK, PERK-eIF2α, and C/EBP homologous protein (CHOP), along with the nuclear factor NF-κB.

AA was studied with the CREB protein signaling during neuronal differentiation, concluding that in the CREB signaling pathway the expression of 17 genes was significantly changed after exposures to 1 or 70 μM, reporting that AA interferes with important cholinergic and dopaminergic neuronal markers during differentiation, which were downregulated after exposure to 70 μM AA [80].

In other studies [41], the intracellular levels of interleukin 6 (IL-6) and tumor necrosis factor alpha (TNF-α) in SH-SY5Y cells were assessed in order to look into the potential pro-inflammatory effects of AA. The findings showed that, in comparison to the control value, the TNF-α content was significantly raised following treatment with 2.5 and 5 mM AA; however, the level of IL-6 was only significantly increased in the 5 mM AA group when compared to the control group. In addition, nuclear NF-κB and the mitogen activated protein kinases (MAPK) signaling pathway with JNK and p38 were activated by AA. 

The same research group studied the induction of AA in phosphorylated tau aggregation, phosphorylated CREB protein reduction, and Bax/Bcl-2 ratio upregulation in SH-SY5Y cells at the same concentrations reported above. Furthermore, AA induced glycogen synthase kinase-3β (GSK-3β) and upregulated activating transcription factor 4 (ATF4) and CHOP in SH-SY5Y cells. It also activated the protein kinase RNA-like endoplasmic reticulum kinase (PERK)-Eukaryotic Initiation Factor 2 alpha (eIF2α) signaling [42].

However, 1.25, 2.5, and 5 mM of AA were found to increase the cleavage of caspase-3 and the poly ADP ribose polymerase (PARP) enzyme (the initiator and effector caspases in the intrinsic apoptotic pathway, respectively). They also decreased Akt phosphorylation in SH-SY5Y cells, which prevented the activation of the PI3K/Akt pathway [43]. GSH levels were restored and H_2_O_2_ stimulation was inhibited by LA pretreatment, which counteracted AA-induced alterations in GSH loss and H_2_O_2_ production. The downregulation of AMP-activated protein kinase (AMPK) and glycogen synthase kinase-3 beta (GSK3β) phosphorylation were induced by AA in a dose-dependent manner; AA elicited energy deficits in the SH-SY5Y cells by regulating the AMPK/GSK3β cascade, resulting in Ca^2+^ disturbance, ATP depletion, and CREB/BDNF signaling impairment. In addition, Sirtuin 1 (Sirt1) and peroxisome proliferator-activated receptor γ co-activator 1 α (PGC-1α) were downregulated by AA; however, AA elicited cellular autophagy in terms of the conversion of microtubule-associated protein light chain 3 (LC3)-I to LC3-II and beclin-1 protein expression. Also, AA induced the overexpression of prostaglandin-endoperoxide synthase 2 (COX-2) and inducible nitric oxide synthase (iNOS), and AA reduced the phosphorylation of extracellular signal-regulated kinases (ERK) while intensifying the phosphorylation of JNK and p38 in MAPKs. In addition, AA mediated a reduction in nuclear factor erythroid 2–related factor 2 (Nrf2) nuclear translocation and cytoplasmic Kelch-like ECH-associated protein 1 (Keap1) expression, resulting in the downregulation of the expression of phase II enzymes, namely HO-1 and NAD(P)H Quinone Dehydrogenase 1 (NQO1) [43].

In brief, AA modulated several signaling pathways related to differentiation, inflammation, and apoptosis, disturbing transcriptomic factors such as nuclear NF-κB and MAPKs signaling pathway with JNK and p38 activation, COX-2 and iNOS overexpression, and AMPK and GSK3β downregulation. AA also inhibited the activation of the PI3K/Akt pathway and downregulated Sirt1 and PGC-1α. Furthermore, it interfered with the CREB signaling pathway, an important cholinergic and dopaminergic neuronal marker during SH-SY5Y differentiation (Table 8).

## 7. Gene Expression

### 7.1. Acrylamide (AA)

The impact of AA on gene expression, as elucidated by recent research, highlights a complex and nuanced response within cellular systems. A comprehensive analysis of differentially expressed genes (DEGs) following exposure to AA revealed distinct patterns of gene dysregulation.

Following exposure to 70 µM AA over a six-day differentiation period, a notable dysregulation was observed in a set of eight genes. Among these genes, NTRK2 (Neurotrophic Receptor Tyrosine Kinase 2, a receptor of BDNF), FGF1, RASD2, BMP7, SEMA3F (Semaphorin 3F), and SEMA5A (Semaphorin 5A) exhibited significant alterations, indicating potential pathways affected by AA-induced toxicity during cellular differentiation [81]. In the same study, they also studied other compounds; therefore, the shared dysregulation of CNR1 (encoding cannabinoid receptor 1) and SEMA5A between AA and valproic acid exposures, as well as OPRD1 (encoding opioid receptor delta 1) between AA and rotenone, underscores the intriguing overlap in genetic response among these neurotoxic compounds [81].

In addition to examining markers linked with CREB signaling pathways, Attoff et al., 2020 [81] opted to incorporate indicators associated with cholinergic (CHAT) and dopaminergic (DRD2 and MAOA) neurons in consideration of their crucial role in the neuronal differentiation process of SH-SY5Y cells. They also included synaptotagmin 1 (SYT1), known for its protein adduct formation with AA, along with genes previously impacted by AA exposure (such as MAOA and FGF1). Additionally, BDNF and tropomyosin receptor kinase B (TrkB), closely associated with the CREB signaling pathway, were part of the selection. The analysis revealed a notable decrease in the expression of 6 out of 16 genes following exposure to 70 μM of AA, with 5 of them demonstrating decreased expression even after exposure to 1 μM of AA. Interestingly, the expression of CHAT, TGFB1, STXBP2, BDNF, and DRD2 showed an increase during differentiation compared to undifferentiated cells. However, these genes were all downregulated upon exposure to AA. Notably, while MAOA demonstrated an increased expression compared to undifferentiated cells, it exhibited significant reduction only after exposure to 70 μM of AA [80].

### 7.2. Deoxynivalenol (DON)

For DON, Kalagatur et al. [63] studied the ability of these mycotoxins to alter the expression of significant neuronal markers was analyzed, reporting a significant downregulation at 50 and 100 µM of BDNF, AADC, and TH mRNA expression.

### 7.3. Ochratoxin A (OTA)

Similarly, but at higher concentrations of OTA (130–0.20 nM) and at 7 days of exposure, an alteration in several cell cycle (p21, p53, cyclin B and D) and neuronal differentiation (GAP43, Wnt5a and TUBB3) genes was found [71]. Concerning the gene and protein expression, several genes and proteins were studied, all of them implicated in the cell proliferation (BAX, P53, MAPT, TPPP p21, cyclin B and D, GAP43, Wnt5a, and TUBB3). At the highest dose studied, 1 µM OTA exposure for two days reduced the expression of P53, BAX, and MAPT mRNA at Days 1 and 2. In comparison to the control exposure groups, TPPP mRNA expression rose on Day 2 after exposure to 1 μM OTA but decreased on Day 1 [82]. The duration of exposure and the relationship between the duration of exposure and the 1 μM OTA dose were indicated by the expression of BDNF mRNA. For 11 days, cells exposed to lesser doses (2 fM, 20 fM) showed no change in gene expression [71].

### 7.4. T-2 Toxin 

To investigate the impact of the T-2 toxin on mitochondrial biogenesis in SH-SY5Y cells, researchers measured the mtDNA copy number and the expression levels of NRF2, KEAP1, PGC-1α, NRF1, and TFAM [69]. For cells treated with 5 and 10 ng/mL, the mtDNA copy number decreased to 80.3% and 60.9%, respectively. The expression of NRF2 mRNA increased to 1.7 and 2.8 times, respectively [69]. Conversely, KEAP1’s mRNA expression dropped to 45.4% and 77.0%. The T-2 toxin significantly reduced the expressions of PGC-1α and its downstream targets NRF1 and TFAM. The most notable reductions were observed in the gene levels, which were lowered by 5 and 10 ng/mL of T-2 toxin, respectively, to 56.3% and 21.6% [69].

### 7.5. Zearalenone (ZEA)

When the concentration of ZEA increased in the comet assay, the tail dispersion increased in a dose-dependent manner. Additionally, a substantial increase in apoptotic nuclei was observed in treated cells compared to control cells at 12 and 24 h after exposure. This “ladder” pattern is indicative of oligonucloesomal DNA fragmentation. Additionally, BDNF and TH mRNA expression was not changed and gradually declined with an increase in toxin concentration for 12 and 24 h after ZEN exposure when gene expression was examined at the lower dose of ZEA (25 µM). Conversely, at 25 µM, AADC mRNA expression was inhibited. Further suppression of AADC mRNA expression was achieved by increasing the concentration of ZEA toxin (50 and 100 µM) [70]. In the case of the metabolites, other genes were studied and similar results were obtained; in α-ZEL treated cells, the mRNA of CASP3 and BAX mRNA were overexpressed at 12.5 and 25 μM. And in the case of β-ZEL, it upregulated Erβ mRNA at 12.5 μM while it downregulated the expression of CASP3 and BCL2 [75].

### 7.6. Fumonisin B1 (FB1)

Finally, for FB1, the ability to produce DNA damage was studied, through the comet assay, TUNEL assay, and DNA fragmentation assay. In the comet assay and when compared to other treatments, DNA damage was the highest 48 h after FB1 treatment, as indicated by an increased tail length and a decreased head diameter. Furthermore, compared to the control, considerable DNA damage resulted from a 24 h toxin treatment. Additionally, TUNEL assay results showed that FB1 treatment for 12, 24, and 48 h increased TUNEL-positive cells relative to the control [65]. In concordance, DNA ladders were seen after treatment with 30 and 100 µM FB1 for 48 h [77].

To sum up, mycotoxins have demonstrated the capacity to modulate the gene expression in SH-SY5Y cells, as well as to produce DNA damage (Table 9).

## 8. Protein Expression

### 8.1. Acrylamide (AA)

Although the study of transcription factors is important to know which signaling pathways can be altered, the importance lies in the final expression or suppression; this is why the protein content and how the presence of AA modulates it was considered. 

According to the signaling pathways and transcriptomic factors altered as mentioned above, the proteins involved in processes of stress, neuronal differentiation, inflammation, and cell death were analyzed, such as heat shock proteins (HSPs), microtubule-associated proteins (MAPs), focal adhesion kinase (FAK), and CHOP.

In this sense, on one hand, it has been proved that the treatment of cells with AA increased the cell death through the caspase-3 activity and an increase in cells in the sub-G1 phase. Both caspase-3 activation and the sub-G1 cell population peaked when the cells were exposed to 3 mM AA. Interestingly, a higher dose of AA (4–5 mM) resulted in less caspase-3 activity, even though the cytotoxicity increased. This indicates that the decrease in caspase-3 activity at 5 mM of AA is not solely due to heightened cytotoxicity [48].

One year later, the same research team found that, after exposing human neuroblastoma cells (SH-SY5Y) to 0.5–5 mM AA for 18 h, the levels of HSPs of 90, 70, and 27 kDa (Hsp90, Hsp70, and Hsp27, respectively) were elevated in the incubation media depending on the dose of AA, while only the Hsp70 level increased within cells [83].

According to other work, exposure to AA predominantly causes eukaryotic translation initiation factor 2α (eIF2α) to be phosphorylated. This is followed by the buildup of ATF4, the protein that binds to eIF2α. Additionally, it was demonstrated that CHOP mRNA expression dramatically increased at all investigated time points and doses, with a notable increase of up to 100-fold at 7.5 or 10 mM AA following an 8 h exposure. Conversely, there was no alteration on the protein levels of GRP94, glucose-regulated protein (GRP78), or endoplasmic reticulum (ER) chaperones. These results imply that the AA exposure of SH-SY5Y cells causes the production of the pro-apoptotic CHOP protein but not of the ER chaperones that provide cytoprotection [46].

The effects of AA on the p53 protein and intracellular signal transduction pathways were examined using human neuroblastoma SH-SY5Y cells. p53, phosphorylated p53, and p53-associated protein murine double minute 2 (MDM2) were all upregulated by AA. The Ser15 position was the only location where p53 was phosphorylated. Extracellular signal-regulated protein kinase (ERK) and p38 were phosphorylated by MAPKs at increasing AA doses (0.5–5 mM), but not c-Jun NH2-terminal kinase [48].

Numerous proteins are impacted by AA-induced adduct formation, including SNAP-25 (synaptosome-associated protein) and other proteins found in synaptic vesicles [8]. In order to confirm the decrease in axon quantity when AA was present, cocultures were labelled with antibodies against both III-tubulin and the pan-neuronal marker PGP 9.5 [49]. The number of axon profiles in response to AA decreased in both instances, and the results matched the information obtained from SNAP-25 immunocytochemistry. 

According to the findings of other research groups’ in vitro studies, activating SphK1 in SH-SY5Y cells also controlled MAPK signaling. This included raising the phosphorylation of extracellular signal-regulated protein kinases (ERK) and lowering that of JNK and p38 [71]. These findings imply that SphK1 activation can provide nerve cell protection against AA-induced damage. 

MAPs are responsible for the polymerization, stabilization, and dynamics of the microtubule network; it has been detected that the expressions of MAP1b and MAP2c in SH-SY5Y cells were reduced by 39% and 64%, respectively, when they treated the cells with 2 mM of AA [44], while the expressions of MAP1b and MAP2c in U-1240 MG cells were reduced by 52% and 57%, respectively. In terms of Janus kinase (JAK)-signal transducer and activator of transcription (STAT) signaling, JAK1 expression was increased by 93% in 10 μM retinoic acid (RA)-stimulated SH-SY5Y cells and by 108% in 10 μM butyric acid (BA)-stimulated U-1240 MG cells. Moreover, JAK1 expression in U-1240 MG cells was reduced by 74% when treated in combination with 10 μM BA and 2 mM AA, whereas JAK1 expression in SH-SY5Y cells was reduced by 68% when treated in combination with 2 mM AA and 10 μM RA. These findings demonstrated that in SH-SY5Y and U-1240 MG cells, AA-inhibited differentiation was mediated through MAPs expression and JAK-STAT signaling, arguing that exposure to AA inhibits cellular differentiation in human neuroblastoma and glioblastoma cells and that downregulation of the JAK-STAT signal pathway may contribute to the understanding of AA-induced neurodegeneration [44].

The differentiating process of neuroblastoma SH-SY5Y cells has also been studied with AA inducing time-dependent tyrosine phosphorylation of FAK [pY^397^] and Pyk2[pY^402^] [78].

Shortly, we can conclude that the expressions of MAP1b and MAP2c, typically involved in microtubule networks, were reduced in SH-SY5Y and U-1240 MG cells which indicated a clear impairment of cell shutdown upon AA exposure. In addition, a significant pro-inflammatory response with JAK1 overexpression was observed. Similarly, in vitro transcriptomic analysis showed how AA exposure (2.5–5 mM) induced apoptosis (increased levels of p53, MDM2, and caspase-3 expression), oxidative stress (ATP4 and CHOP overexpression), and inflammation with high levels of IL-6 and TNF- α. It is also necessary to underline the downregulation of SphK1 which is associated with inflammation and antiapoptotic processes (Table 10).

### 8.2. Mycotoxins 

#### 8.2.1. Fumonisin B1 (FB1)

Other studies have evaluated the capacity of this mycotoxin to induce apoptosis; therefore, some authors investigated the protein expression alterations (caspase 9 and 3, p53, Bax, Bcl-2, Bcl-XL, and Mcl-1) when FB1 was applied to SH-SY5Y cells. Initially, exposure to FB1 did not result in significant alterations in caspase-3-like protease activity. However, after a full day, 100 µM FB1 caused a slight but statistically insignificant increase in caspase-3 activity. During either FB1 treatment period, there was no caspase-3 or caspase-9 cleavage. Exposure to 100 µM FB1 for 72 h also did not affect the amounts of p53 in nuclear or cytoplasmic extracts. These findings are consistent with earlier research that did not discover a statistically significant rise in cell mortality at the concentrations examined (0.1–30 µM) [61,65,77].

On the other hand, in comparison to the control cells, FB1 had no effect on the expression of the pro-apoptotic protein Bax at any of the doses or time points. Furthermore, in SH-SY5Y cells, FB1 had no effect on the investigated anti-apoptotic proteins (Bcl-2, Bcl-XL, and Mcl-1) [85]. Another study suggested that mitochondria may be involved in FB1-mediated cell death because Bax expression rose 48 h after FB1 treatment in comparison to the control, whereas cytochrome C expression increased 24 h after FB1 treatment [61]. After exposure of neuroblastoma cells to FB1, there was a significant increase in JNK phosphorylation, which peaked at 48 h. Additionally, there was a significant increase in the expression of IRE1-α and PERK. Lastly, there was a significant activation of CHOP after 24 h of FB1 exposure [65].

#### 8.2.2. Ochratoxin A (OTA)

In contrast, OTA treatment in SH-SY5Y cells activated caspase-9 and caspase-3 in one study, while another study did not detect activated caspase-3 or changes in p53 phosphorylation. This suggests that OTA-induced cytotoxicity may have different mechanisms at play [68,78].

#### 8.2.3. T-2 Toxin

As for the T-2 toxin, it led to an increase in NRF2 protein expression, potentially indicating an adaptive response to oxidative stress induced by the toxin [69]. 

Regarding mycotoxins, FB1 significantly increased the phosphorylation of JNK in neuroblastoma cells, as well as significantly increasing the expression of CHOP, IRE1-α, and PERK. OTA treatment in SH-SY5Y activated caspase-9 and caspase-3 in one study but not in another. Finally, the T-2 toxin increased NRF2 protein expression. Therefore, more studies are needed in order to state that FB1, OTA, and the T-2 toxin lead to a modulation in protein expression in SH-SY5Y cells (Table 11).

## 9. Natural Bioactive Compounds 

For all of the above, we can conclude that AA produces toxicity in several neuronal cell lines in a dose- and time- dependent manner; nevertheless, some authors have shown that besides AA toxicity, some natural compounds may reduce this effect on neuronal cells. Pre-treatment with 6 µM curcumin was found to considerably reduce the neuronal toxicity caused by 2.5 mM AA. This was demonstrated by improved cell viability, reduced levels of intracellular ROS and MDA, and increased levels of GSH. Additionally, curcumin pre-treatment eliminated aberrant tau phosphorylation, P-CREB decrease, and CHOP-induced apoptosis in SH-SY5Y cells, further lowered GSK-3β and ATF4 activity, and blocked PERK-dependent eIF2α phosphorylation [42]. In this same line, but with another compound, Chen and Chou 2015 [44] demonstrated that 0.25, 0.5, and 1 mM of caffeine attenuated 2 mM AA-inhibited phosphorylation of MAPKs in U-1240 MG cells. In addition, 50 µM of Z-VAD-fmk, a pan-caspase inhibitor, could be a protective compound against 0 to 5 mM of AA by abolishing caspase-3 activities in cells exposed to AA, lowering LDH leakage and increasing cell viability [50].

One step further, and in order to obtain more accurate results, Ning et al., 2021 [73], showed that 0.014 to 10 µg/mL of triphala, a herbal mixture from India, suppressed 10 mM AA-induced neurotoxicity and scavenged free radicals in a zebrafish model, suggesting that triphala could be a potential agent to treat neurodegenerative diseases associated with oxidative stress (Table 12).

Regarding mycotoxins and possible strategies to mitigate the cytotoxicity of this compound, several studies have reported the cytoprotective capacity of some bioactive compounds, such as *Allium sativum* L. garlic against BEA and ZEA metabolites [60], coffee by-products against BEA [58], NAC against FB1 [65], lutein, zeaxanthin, and goji berries extract against BEA [62], and beetroot extract against FB1 and OTA [67] (Table 12).

## 10. Discussion 

**Figure 2:**
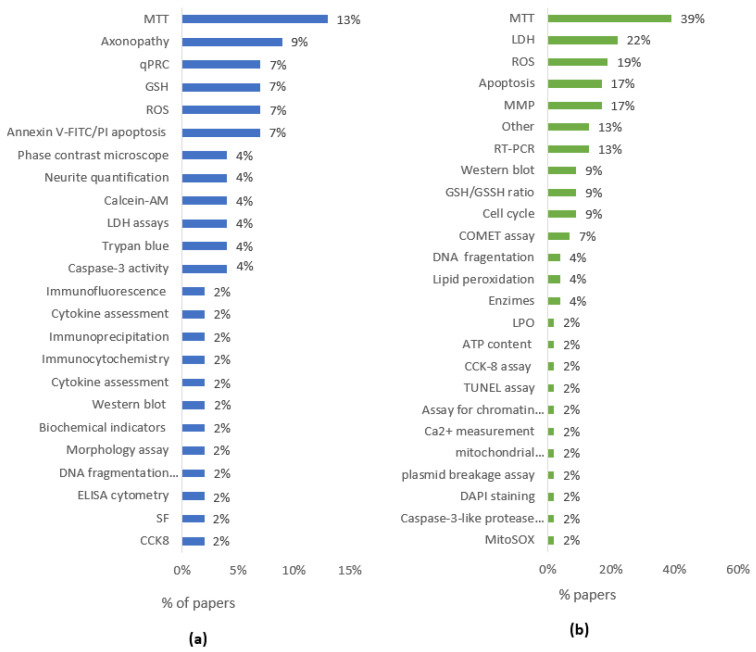
(**a**) Percentage of employed assays in SH-SY5Y cells treated with AA according to publications reviewed (n = 27). (**b**). Percentage of employed assays in SH-SY5Y cells treated with mycotoxins according to publications reviewed (n = 36).

Overall, all AA studies were carried out in SH-SY5Y, except two that used U-1240 MG and PC12 cells, the range of concentrations went from 0.1 to 10 mM, and to highlight the techniques employed the most widely used was the MTT (13%), followed by the axonopathy assay (9%) and qPCR, Annexin V-FITC/PI apoptosis, GSH, and ROS assays (7% in all cases). Caspase-3 activity, Trypan blue, LDH assays, Calcein-AM, Neurite quantification, and phase-contrast microscopy were used in a proportion of 4% each. Finally, CCK8, SF, ELISA cytometry, DNA fragmentation detection, morphology assay, biochemical indicators, Western blot, immunocytochemistry, immunoprecipitation, immunofluorescence, and cytokine assessment were used in proportions of 2% for each one (Figure 2a).

Regarding mycotoxins, the main cells studied where SH-SY5Y, and the range of concentrations went from 0.009 to 200 µM. The most used techniques were MTT (39%), LDH (22%), ROS (19%), apoptosis, and MMP (17% in both cases), followed by RT-PCR (13%), Western blot, GSH/GSSH ratio, and cell cycle (9% in all cases). DNA fragmentation, lipid peroxidation, enzymes activity (4%), and the rest of the assays performed in SH-SY5Y cells were used in proportions of 2% for each one (Figure 2b).

As for the main MoA studied in SH-SY5Y cells treated with AA, cytotoxicity reached 32%, followed by axonopathy with a 20%, and the study of protein expression achieving 14% of the total of studies.

Concerning the mycotoxins, the main MoA studied in SH-SY5Y cells included cytotoxicity which reached 30%, mitochondrial membrane potential alterations at 16%, and oxidative stress at 15%.

On the other hand, several bioactive compounds were tested to modulate AA toxicity in this review; the use of curcumin, caffein, Z-VAD-fmk, and triphala was reported. 

The most studied mycotoxin in SH-SY5Y cells was BEA, followed by ZEA and its metabolites, OTA and FB1.

## 11. Conclusions

Overall, we could conclude that the common toxic effects reported for both AA and several mycotoxins highlight an interesting field to investigate, not only because of their presence in the diet but also for the possibility of the merged effect on human health and its consequences at the neuronal level. Although there are several assays which report toxic effects, for these compounds the most used assays were MTT followed by axonopathy and qPCR, the less frequent assays were ELISA, SF, and CCK8, among others in the case of AA. For mycotoxins, MTT, LDH, ROS, apoptosis, MMP, and RT-PCR were the most employed techniques and MitoSOX, DAPI staining, Ca^2+^ measurement, and CCK-8 assay, among others, were less frequently used. The MoA reported were cytotoxicity, axonopathy, protein expression, apoptosis, increased cellular reactive oxygen species, and a reduction in intracellular GSH, impaired neurite outgrowth during differentiation in SH-SY5Y, and other neuronal cells, in a time- and concentration-dependent manner for AA and cytotoxicity, oxidative stress, gene expression, apoptosis, protein expression, cell cycle disruption, and genotoxicity in a in a time- and concentration-dependent manner for mycotoxins. Furthermore, similar to mycotoxins, AA modulated signaling pathways related to inflammation, oxidative stress, apoptosis, and differentiation through changes in the expression of proteins involved in different processes.

Although AA and mycotoxins neurotoxicity has been widely reported in the literature, several bioactive compounds naturally present in food products could reduce the toxic effects of AA and mycotoxins, according to the literature. On one hand, curcumin, caffein, Z-VAD-fmk, and triphala, tested in a similar percentage, have been shown to mitigate AA toxicity. On the other hand, *Allium sativum* L. garlic, coffee by-products, NAC, lutein, zeaxanthin, goji berries, and beetroot extract have shown to cytoprotect SH-SY5Y cells against mycotoxins like BEA, OTA, and FB1. Nevertheless, more in vivo and in vitro studies are needed to better investigate not only AA and mycotoxins’ neuronal damage but also the possible beneficial role of bioactive compounds and how this mechanism of reduction in damage is happening.

## 12. Materials and Methods

For this review, a standard systematic literature search in PubMed, Scopus, and Web of Science was conducted by including the terms “acrylamide”, “mycotoxins”, “OTA”, “DON”, “BEA”, “ZEA”, “FB1”, “ENN”, “T-2”, “neurotoxicity”, “toxicity” “neuroblastoma”, “in vitro”, and “SH-SY5Y”. The application of these search terms aimed to cover most of the literature regarding the study of the AA neurotoxicity research in the SH-SY5Y cell line. In order to discard unnecessary, incomplete, or irrelevant literature, the abstracts of the reports obtained were assessed. The search retrieved 97 articles, of which 59 were original, accessible, and AA-specific papers and thus were included in the analysis. The exclusion criteria for the remaining 38 articles were associated to the following: (i) written in a language different from English and/or not available online, (ii) representing a review, or (iii) not specific for AA and mycotoxins. 

## Figures and Tables

**Table 1:** Cytotoxicity in SH-SY5Y cells. AA exposure conditions, assays, and effects.

Dose	Exposure Time	Assays	Effects	References
1 to 10 mM	8 h	Cell Count Reagent SF	At 10 mM AA	[46]
1–5 mM	16 h, 20 h	Caspase-3 activity	At 1–5 mM	[45]
2.5, 5, 7.5, and 10 mM	24 h	CCK8	Reduction of 14%, 35%, 48%, and 61%, respectively	[42]
0.8, 20 and 500 μg/mL	24 h	MTT	Reduction of 60% and 70% at the higher concentrations	[55]
2, 2.5, 3, 4 and 5 mM	24 h	MTT	Reduction of 10%, 18%, 20%, 25%, and 30%	[41]
1.25, 2.5, 5 mM	24 h	MTT	Significant loss of cell viability	[43]
0–500 mM	24 h	CellTiter-Blue Cell Viability Assay	IC_50_ 5 mM NI_20_ 0.26 mM	[52]
1, 10 and 100 μM	24 h	MTT, ATP, Caspase	100 μM reduced cell viability	[56]
0.01 mM to 12 mM.	24 h	Propidium iodide and calcein-AM	0.5–2.0 mM of AA was not cytotoxic; 4 mM, produced a significant loss of HuD-positive neurons (58 ± 7%), and the number of axons decreased to 21 ± 5%	[42]
0.5–5 mM	24 h	Trypan blue	Cytotoxic at 5 mM	[48]
0.1–10 mM	24 h	LDH assays	AA was cytotoxic at 10 mM	[49]
0.1–1000 μM	48 h	Calcein-AM, βIII-tubulin, and LDH assay	10% reduction at 1 mM	[54]
0–1000 µM	72 h	Total protein content	>1000 μM IC_50_	[52]
0, 0.5, 1, 2, 5, and 10 mM	0, 6, 12, 24, 48, and 72 h	Trypan blue and LDH	10 mM reduced 38% cell viability at 6 h	[47]
0, 0.1, 0.5, 1, and 2 mM	24, 48, 72 h	MTT	Reduction of 20%, 30%, and 50%	[44]
1–1000 μM.	24, 48, and 72 h; 3, 6, and 10 days.	BradUrd, Sub-G_1_, ^3^H-thimidine, and MTT	Reduction of 30% (48 h) and 90% (72 h) At 100 μM or higher viability decreased at Day 3 and 6.	[45]

AA: acrylamide, ATP: adenosine triphosphate, CCK8: Cell counting kit-8, IC_50_: 50% inhibition concentration, LDH: lactate dehydrogenase, MTT: (3-[4,5-dimethylthiazol-2-yl]-2,5 diphenyl tetrazolium bromide), NI_20_: 20% reduction in network formation.

**Table 2:** Cytotoxicity in SH-SY5Y cells. Mycotoxin, exposure conditions, assays, and effects.

Mycotoxin	Dose	Exposure Time	Assays	Effects	References
**DON**	0–10 µM	6 h and 24 h	MTT LDH	IC_50_: 2.25 µM at 24 h; 1.5 at 48 h Not cytotoxic	[61]
	0–200 µM	6 h and 24 h	MTT LDH	IC_50_: 120 µM at 24 h	[63]
**BEA**	0.009–25 µM	24 h, 48 h, and 72 h	MTT assay	BEA IC_50_: 2.5 at 72 h	[59]
	0.08–2.5 μM	24 h and 48 h	MTT assay	Decrease of 43% in cell viability at 2.5 μM after 48 h	[60]
	0.08–2.5 µM	24 h and 48 h	MTT assay	IC_50:_ 2.5 μM at 72 h	[58]
	0.08–25 µM	24 h and 48 h	MTT assay	IC_50_: 3.2 μM at 24 h; 5 μM at 48 h	[62]
	0.1–30 µM	6 h, 24 h, and 48 h	MTT assay LDH assay	IC_50_: 1.9 µM for 6 h, 1.7 µM for 24 h, and 1.5 µM for 48 h Inducing LDH leakage at 1 μM	[61]
	0–2.5 µM	24 h, 48 h, and 72 h	MTT assay	IC_50_ at 72 h was 2.5 µM	[57]
**ZEA α-ZEL and β-ZEL**	0.39–100 µM	24 h, 48 h, and 72 h	MTT assay	IC_50_: α-ZEL: 20.8 at 48 h, 14.0 at 72 h β-ZEL: 94.3 at 24 h, 9.1 at 48 h, and 7.5 at 72 h	[59]
	0.4–12.5 μM	24 h and 48 h	MTT assay	β-ZEL: 6.25, 12.5, and 25 μM from 31% to 82%	[60]
	0.1–30 µM	6 h, 24 h, and 48 h	MTT assay LDH assay	ZEA presented a IC_50_ of 17.4 μM LDH leakage at 20 µM (9.1 ± 8.9%) and 30 μM (19.5 ± 4.3%) at 24 h	[61]
	25, 50, 75, 100 and 200 µM		MTT assay LDH	Decreased by 86% at 200 µM	[70]
**FB1**	0.1–30 µM	6 h, 24 h, and 48 h	MTT assay LDH assay	At 30 µM after 48 h LDH not cytotoxic	[61]
	50 µM	12 h, 24 h, and 48 h	LDH assay	FB1 led to LDH release at 12 h	[65]
**ENN’s**	0.1–30 µM	6 h, 24 h, and 48 h	MTT assay LDH assay	ENN A IC_50_ of 2.4 µM at 6 h and 2.25 µM at 24 h ENN B IC_50_ of 0.43 μM at 24 h ENN A increased LDH at 5 and 10 μM at 6 h and 24 h ENN B showed LDH release of 20% at 0.25 μM after 24 h	[61]
**OTA**	1, 10, or 100 µM	30, 60 min, 24 h,	LDH	LDH at 1, 10, or 100 µM were 128% ± 1%, 125% ± 2%, and 200% ± 1%	[68]
	0.2–50 μM	24 h and 48 h	MTT assay	IC_50_: 9.1 μM at 24 h; 5.8 μM at 48 h	[67]
	130–0.20 nM	24 h, 48 h, and 72 h 7 days	MTT assay	No IC_50_ was reached	[71]
**T-2**	5–20 ng/mL T-2	1–48 h	CCK-8 assay LDH assay	Viability reduced 81.9%, at 5 ng/mL, 40.8% at 10 ng/mL, and 35.5% at 20 ng/mL LDH levels were increased by 1.8, 2.9, and 3.2 times	[69]

BEA: Beauvericin, CCK8: Cell counting kit-8, DON: Deoxynivalenol, ENN’s: enniatin, FB1: Fumonisin B1, IC_50_: 50%inhibition concentration, LDH: lactate dehydrogenase, MTT: (3-[4,5-dimethylthiazol-2-yl]-2,5 diphenyl tetrazolium bromide), OTA: Ochratoxin A, ZEA: Zearalenone, α-ZEL: alpha-zearalenol, β-ZEL: beta-zearalenol.

**Table 3:** Apoptosis in SH-SY5Y cells. AA exposure conditions, assays, and effects.

Dose	Time	Assays	Effects	References
2.5 mM	24 h	Annexin V-FITC/PI apoptosis	The early apoptotic rate was 5.5%, the late apoptotic rate was 11.8%	[42]
2.5 mM	24 h	Annexin V-FITC/PI apoptosis	Induced apoptosis	[41]
0.5–10 mM	0–72 h	DNA fragmentation detection (mono and oilgonucleosomes)	DNA fragments increased	[47]
1.25–2.5 mM	24 h	Annexin V-FITC/PI apoptosis	Increased with increasing AA concentrations	[72]
10 mM	24 h	ELISA and cytometry	AA induced neuroapoptosis in zebrafishes and produced free radicals	[73]

AA: acrylamide, DNA: deoxyribonucleic Acid, JNK: c-Jun N-terminal kinase, NF-κB: nuclear factor κB, SH-SY5Y: neuroblastoma cell line.

**Table 4:** Apoptosis in SH-SY5Y cells. Mycotoxins exposure conditions, assays, and effects.

Mycotoxin	Dose	Exposure Time	Assays	Effects	References
**DON**	0–10 µM	6 h and 24 h	Cell cycle, FC	Did not produce cell death	[61]
	0–200 µM	6 h and 24 h	Apoptosis by FC	Induced apoptotic nuclei formation	[63]
**BEA**	2.5, 1.25, 0.78 and 0.39 μM	24 h and 48 h	Cell cycle Apoptosis/necrosis	Cell cycle arrest at G0/G1; Apoptotic/necrotic cells increased up to 89% for 24 h and up to 38.8% for 48 h	[74]
	0.1–30 µM	6 h, 24 h, and 48 h	FC analysis (Apoptosis)	Increase of 55.9 ± 8.6%. in apoptotic cells	[61]
**ZEA α-ZEL and β-ZEL**	12.5, 6.25, 3.12, and 1.56 μM	24 h and 48 h	Cell cycle Apoptosis/necrosis	Concentration-dependent increase in G0/G1 phase at 24 h; α-ZEL increased apoptotic and apoptotic/necrotic cells at 24 h; β-ZEL increase apoptotic cells exposed to 12.5 μM after 24 and 48 h	[73]
	0.1–30 µM	6 h, 24 h, and 48 h	FC analysis (Apoptosis)	Did not produce cell death	[61]
**FB1**	0.1–100 µM	48 h, 72 h, and 144 h	Cell viability (propidium Iodide)	Viability decreases at 100 µM at 48–144 h	[64]
	0.1–30 µM	6 h, 24 h, and 48 h	FC analysis (Apoptosis)	Did not produce cell death	[61]
	50 µM	12 h, 24 h, and 48 h	Apoptosis assay (FC)	Induced apoptosis	[65]
**ENN’s**	0.1–30 µM	6 h, 24 h, and 48 h	FC analysis (Apoptosis)	Increase in apoptotic cell death of 49.2 ± 8.2% at 24 h and 46.0 ± 9.3% at 48 h	[61]
**OTA**	130–0.20 nM	24 h, 48 h, and 72 h 7 days	Cell cycle (FC)	Low OTA doses altered neuronal differentiation and cell cycle	[71]

BEA: Beauvericin, DON: Deoxynivalenol, ENN’s: enniatin, FB1: Fumonisin B1, FC: flow cytometry, OTA: Ochratoxin A, ZEA: Zearalenone, α-ZEL: alpha-zearalenol, β-ZEL: beta-zearalenol.

**Table 5:** Oxidative stress. AA dose, exposure time, assays, effects, and references.

Dose	Time	Assays	Effects	References
10 mM	6 h	ROS	Induces ROS	[46]
2.5 mM and 5 mM	24 h	GSH, MDA, and ROS	2.5 and 5 mM decreased intracellular GSH production and increased MDA and ROS generation	[41]
2.5 mM	24 h	ROS (DCFH-DA) and LPO (MDA, GSH)	Maximal ROS production; 2.5 mM (+40%) MDA content: +100% 2.5 GSH content: −50% 2.5 mM	[42]

**DCFH-DA:** Dichloro-dihydro-fluorescein diacetate, **GSH:** glutatión, **LPO:** lipid peroxidation, **MDA:** malondialdehyde, **ROS:** reactive oxygen species.

**Table 6:** Oxidative stress. Mycotoxins dose, exposure time, assays, effects, and references.

Mycotoxin	Dose	Exposure Time	Assays	Effects	References
**DON**	0–200 µM	6 h and 24 h	ROS generation by fluorometry LPO and antioxidant enzyme levels MMP	ROS generation for 6 and 24 h Induction of LPO and exhaustion of antioxidant enzymes Induction of MMP loss	[63]
**BEA**	2.5, 1.25, 0.78, and 0.39 μM	2 h, 24 h, and 48 h	ROS GSH	ROS decrease at 1.25 and 2.5 μM, from 45 to 120 min; GSH/GSSG ratio increased from 103% to 142% after 24 h	[75]
	2.5, 1.25, 0.78, and 0.39 μM	24 h and 48 h	Glutathione peroxidase activity (GPx) Glutathione S-transferase (GST) activity Catalase (CAT) activity Superoxide dismutase (SOD) activity	GPx activity, BEA promoted a 9- to 17-fold increase after 24 h and a 2- to 9-fold for 48 h; GST activity, a slight increase (4–32% at 48 h) was obtained, while for SOD, a 2.5-fold increase at 48 h was shown; No significant differences for CAT were observed	[76]
	0.1–30 µM	6 h, 24 h, and 48 h	MMP	Alteration of mitochondrial membrane at 2.5–10 μM during 6–24 h	[61]
**ZEA α-ZEL and β-ZEL**	25, 12.5, 6.25, and 3.12 μM	120 min, 24 h, and 48 h	ROS GSH	α-ZEL: at 25 μM increase from 5 to 60 min; β-ZEL: decrease at 12.5 μM from 5 to 90 min and at 25 μM from 15 to 30 min and from 60 to 120 min; GSH/GSSG ratio increased after 24 h at all concentrations from 111% to 148%, for α-ZEL and from 68% to 131% for β-ZEL	[75]
	12.5, 6.25, 3.12 and 1.56 μM	24 h and 48 h	Glutathione peroxidase activity (GPx) Glutathione S-transferase (GST) activity Catalase (CAT) activity Superoxide dismutase (SOD) activity	GPx activity increased by 13.5- to 23-fold for α-ZEL and 9- to 17-fold for β-ZEL. For 24 h and 48 h; GST increased after 48 h of exposure to β-ZEL at 3.12 and 12.5 μM by 22% and 102%, respectively; CAT activity increased from 0.4- to 1.4-fold for α-ZEL and 1–4.2-fold for β-ZEL exposure after 48 h at all concentrations; SOD activity increased for α-ZEL up to 1.4-fold, and for β-ZEL up to 2.5-fold after 48 h	[76]
	0.1–30 µM	6 h, 24 h, and 48 h	MMP	ZEA produced a slight decrease at 30 μM after 24 h of incubation	[61]
	25, 50, 75, 100 and 200 µM		ROS Lipid peroxidation assay MMP	ZEN increased ROS generation, LPO, and loss of MMP in a dose-dependent manner	[70]
**FB1**	0.1–100 µM	48 h, 72 h, and 144 h	ROS Lipid Peroxidation GSH	Did not affect production of ROS; 100 µM increased MDA concentrations after a 24 h, 10 µM increased MDA levels at 72 h, 100 mM decreased GSH levels (61% of controls) at 144 h	[64]
	0.5–200 µM	24 h and 48 h	ROS MitoSOX	0.5, 5, and 50 µM, increased ROS production; All doses increased MitoSOX florescence	[66]
	0.1–30 µM	6 h, 24 h, and 48 h	MMP	No significant changes in ΔΨm	[61]
	50 µM	12 h, 24 h, and 48 h	ROS Mitochondrial superoxide level Ca^2+^ measurement	Increase in ROS production, mitochondrial ROS accumulation, and cellular Ca^2+^ level	[65]
**ENN’s**	0.1–30 µM	6 h, 24 h, and 48 h	MMP	ENN A affected at 5 (79.0 ± 8.9%) and 10 μM (67.7 ± 14.5%,) after 6 h. At 24 h, ENN A affected to ΔΨm at all the concentrations tested; ENN B altered ΔΨm at 5 and 10 μM after 6 h and 24 h	[61]
**OTA**	1, 10, or 100 µM OTA	30, 60 min, 24 h,	ROS	100 µM for 30 and 60 min, indicated the evocation of oxidative stress	[68]
**T-2**	5–20 ng/mL T-2	1–48 h	ROS level, GSH/GSSH ratio, ATP content, MMP	ROS levels significantly increased to 3.8 times at 5 ng/mL and 5.0 times at 10 ng/mL; The ratio of GSH to GSSG decreased to 79.8% at 5 ng/mL and 60.7% at 10 ng/mL; 5 and 10 ng/mL decreased the ΔΨm to 60.7% and 41.5%, while the ATP content decreased to 66.7% and 51.5%	[69]

ATP: adenosine triphosphate, BEA: Beauvericin, DON: Deoxynivalenol, ENN’s: enniatin, FB1: Fumonisin B1, GSH: glutatión, LPO: lipid peroxidation, MDA: malondialdehyde, MMP: Mitochondrial membrane potential, ROS: reactive oxygen species, OTA: Ochratoxin A, ZEA: Zearalenone, α-ZEL: alpha-zearalenol, β-ZEL: beta-zearalenol.

**Table 7:** Axonopathy in SH-SY5Y cells. AA exposure conditions, assays, and effects.

Dose	Time	Assays	Effects	References
0.5 mM or 1 mM	0, 24, 48, and 72 h	Neuronal network integrity (neurites connections)	1 mM AA, at 72 h network levels reduction	[77]
100 nm and 10 pm	3 and 6 days	Phase-contrast microscope	Impairs neurite outgrowth in a time- and dose-dependent manner	[45]
0.8, 20 and 500 μg/mL	24 h	Phase-contrast microscope	Number and the length of processes showed a gradually decreasing tendency	[55]
0.5–2 mM	24, 48, 72 h	Biochemical indicators for morphologic changes;	1 and 2 mM resulted in cells with no neurite morphological extensions	[44]
0.1–1 mmol/L	72 h	Neurite quantification;	ND_20_ 0.21 mmol/L	[52]
0.25 mM	24 h	Axonopathy (neurite degeneration assay	0,25 mM 50% neurite degeneration	[78]
4 mM	24 h and 96 h	Neuronal and axon number	4 mM reduced neuron number by 96 h, to 67.7%	[53]
6 mM	8 h	Axonopathy	Dose-dependent from 2.5 to 10 mM	[79]
250 μM 670 μM	72 h	Axonopathy	Concentration-dependent decrease in the number of neurites/cells. ND_20_: 250 IC_20_: 670	[80]
0.1–10 mM	4 h, 24 h, and 48 h	Morphology assay	Differentiated cells were more sensitive to 1 mM compared with undifferentiated cells	[50]

AA: acrylamide, BDNF: brain-derived neurotrophic factor, IC_20_: concentration causing 20% protein reduction, ND_20_: concentration causing 20% neurite degeneration.

**Table 8:** Signaling pathways in SH-SY5Y cells. AA exposure conditions, assays, and effects.

Dose	Time	Assays	Effects	References
1–70 µM	9 days	qPRC	CREB signaling was downregulated	[80]
0, 1.25, 2.5 and 5 mM	24 h	Cytokine assessment	NF-κB, MAPKs, JNK, and p38 signaling pathway were activated;	[41]
2.5 mM	24 h	Immunofluorescence (tau hyperphosphorylation (pS262))	AA: +50% pS262 Activated the PERK-eIF2α signaling, triggered the activation of GSK-3β, and upregulated ATF4 and CHOP; Also, inhibited the activation of the PI3K/Akt pathway	[42]
1.25, 2.5, 5 mM	24 h	Western blot	Inhibited the activation of the PI3K/Akt pathway; Downregulation of AMPK, Sirt1, PGC-1α, and GSK3β; Phosphorylation by AA in a dose-dependent manner; Overexpression of COX-2 and iNOS	[43]

AA: acrylamide, AMPK: AMP-activated protein kinase, COX: Prostaglandin-endoperoxide synthase, CREB: cAMP response element binding, CHOP: C/EBP homologous protein, GSK-3β: glycogen synthase kinase-3β, iNOS: inducible nitric oxide synthase, JNK: c-Jun N-terminal kinase, MAPKS: mitogen-activated protein kinases, PERK/eIF2α: protein kinase RNA-like endoplasmic reticulum kinase—Eukaryotic Initiation Factor 2 alpha, Pyk2: proline-rich tyrosine kinase 2 RNA (PKR)-like/Pancreatic ER Kinase, PI3K: phosphatidylinositol 3-kinase, PGC-1α: Peroxisome proliferator-activated receptor-gamma coactivator, qPCR: quantitative polymerase chain reaction, Sirt 1: NAD-dependent deacetylase sirtuin-1.

**Table 9:** Gene expression and genotoxicity in SH-SY5Y cells. Mycotoxin dose, exposure conditions, assays, and effects.

Mycotoxin	Dose	Exposure Time	Assays	Effects	References
**DON**	0–200 µM	6 h and 24 h	Comet assay; Gene expression by qRT-PCR	Damage in DNA at 24 h; BDNF and TH mRNA expression were downregulated at 50 and 100 µM; AADC gene expression was downregulated at all concentrations	[63]
**BEA**	2.5, 1.25, 0.78, and 0.39 μM	2 h, 24 h, and 48 h	Gene expression assay by RT-PCR	Upregulated BCL2 mRNA	[73]
**ZEA α-ZEL and β-ZEL**	25, 12.5, 6.25, and 3.12 μM	120 min, 24 h, and 48 h	Gene expression assay by RT-PCR	α-ZEL: mRNA of CASP3 and BAX mRNA were overexpressed at 12.5 and 25 μM; β-ZEL: upregulated ERβ mRNA at 12.5 μM, while downregulated expression of CASP3 and BCL2	[75]
	25, 50, 75, 100, and 200 µM	3 h, 24 h	COMET assay; DAPI staining plasmid breakage assay; Gene expression by qRT-PCR	25 µM increased the tail length by 31% ± 2.6%, and at 100 µM 83% ± 5.3%; Showed DNA fragmentation at 12 h and 24 h; 25 µM of ZEA: BDNF and TH mRNA expression decreased with increased concentration for 12 h and 24 h; AADC mRNA expression was suppressed at 25 µM; The mRNA expression of AADC was further abrogated by 50 and 100 µM	[70]
**FB1**	1, 10, and 100 µM	12 h, 24 h, 48 h, 72 h, and 144 h	DNA fragmentation	DNA ladders were obtained at 30 and 100 µM for 48 h	[76]
	50 µM	12 h, 24 h and 48 h	Comet assay; TUNEL assay	Increased tail length and decreased head diameter time-dependently; At 24 and 48 h, increased TUNEL-positive cells	[65]
**OTA**	2 fM, 20 fM and 2 pM 0.001 μM, 0.01 μM, 0.05 μM, 0.1 μM, 0.5 μM, and 1 μM	2 and 11 days	Gene expression by RT-qPCR	Expression of P53, BAX, and MAPT mRNA were reduced at Days 1 and 2; TPPP mRNA expression decreased at Day 1 at 1 μM	[82]
	0.1, 0.25, 0.5, 1.0 and 2.5 µM	24 h and 48 h	DNA fragmentation assay	Concentration-dependent DNA laddering	[83]
**T-2**	5–20 ng/mL T-2	1–48 h	Quantification of mitochondrial DNA copy number quantification; Gene expression by RT-qPCR	Reduced mtDNA copy number of 80.3% at 5 ng/mL and 60.9%, at 10 ng/mL; NRF2 mRNA expression increased to 1.7-fold at 5 ng/mL and 2.8-fold by 10 ng/mL; The expression of KEAP1 mRNA decreased to 77.0% and 45.4%; The expressions of PGC-1α and its downstream targets NRF1 and TFAM were distinctly inhibited	[69]

BAX: Bcl-2 Associated X-protein, BCL2: B-cell leukemia/lymphoma 2 protein, BDNF: Brain-derived neurotrophic factor, BEA: Beauvericin, CASP3: cysteine–aspartic acid protease, DON: Deoxynivalenol, ENN’s: enniatin, Erβ: Estrogen receptor beta, FB1: Fumonisin B1, KEAP1: Kelch-like ECH-associated protein 1, NRF1: Nuclear respiratory factor-1, NRF2: nuclear factor erythroid 2–related factor 2, OTA: Ochratoxin A, P53: tumor protein p53, PGC-1α: Peroxisome proliferator-activated receptor-gamma coactivator, qPCR: quantitative polymerase chain reaction, TFAM: Mitochondrial transcription factor A, ZEA: Zearalenone, α-ZEL: alpha-zearalenol, β-ZEL: beta-zearalenol.

**Table 10:** Protein expression in SH-SY5Y cells. AA exposure conditions, assays, and effects.

Dose	Time	Assays	Effects	References
1–5 mM	16 h and 20 h		Increased the caspase-3 activity	[48]
0.5–5 mM	18 h	Protein analysis	Hsp90, 70, and 27 levels increased, dose-depending; HSPs increases as the AA grows	[84]
1–10 mM	8 h	qPCR	Induction of the pro-apoptotic CHOP protein expression	[47]
0.5–5 mM	1–24 h	Immunoprecipitation	p53 protein accumulated	[49]
0.01–12 mM.	24, 96, or 144 h	Immunoblotting	30% reduction in SNAP-25-positive axon profiles	[53]
0, 0.1, 0.5, 1, and 2 mM	24, 48, 72 h	Western blot	MAPs expression and JAK-STAT signaling were involved	[45]
6 mM	8 h	Western blot analysis and PCR	FAK and Pyk2 time-dependent tyrosine phosphorylation	[78]

AA: acrylamide, CHOP: C/EBP homologous protein, FAK: focal adhesion kinase, HSPs: Heat shock protein, IC_20_: concentration causing 20% protein reduction, IC_50_: 50% inhibition concentration, JAK-STAT: Janus kinase–signal transducer and activator of transcription, PERK: protein kinase, Pyk2: proline-rich tyrosine kinase 2 RNA (PKR)-like/Pancreatic ER Kinase, qPCR: quantitative polymerase chain reaction, SNAP: synaptic vesicle protein S-nitroso-N-acetyl penicillamine.

**Table 11:** Protein expression in SH-SY5Y cells. Mycotoxin dose, exposure conditions, assays, and effects.

Mycotoxin	Dose	Exposure Time	Assays	Effects	References
**FB1**	1, 10, and 100 µM	12 h, 24 h, 48 h, 72 h, and 144 h	Caspase-3-like protease activity Western blot	No significant changes in caspase-3-like protease activity; 100 µM for 72 h had no effects on p53 levels; Bax Bcl-2, Bcl-XL, and Mcl-1 were not affected.	[85]
**OTA**	1, 10, and 100 µM	30, 60 min, and 24 h	Western blot	Activated caspase-3 was not detected; p53 phosphorylation was not detected	[68]
	0.1, 0.25, 0.5, 1.0, and 2.5 µM	24 h, 48 h	Western blot	Activation of caspase-9 and caspase-3	[78]
**T-2**	5–20 ng/mL	1–48 h	Western blot	NRF2 protein expression increased 1.2-fold by 5 ng/mL and 1.4-fold by 10 ng/mL. The mRNA protein expression decreased to 95.6% and 85.6%	[69]

BAX: Bcl-2 Associated X-protein, BEA: Beauvericin, DON: Deoxynivalenol, ENN’s: enniatin, FB1: Fumonisin B1, NRF2: nuclear factor erythroid 2–related factor 2, OTA: Ochratoxin A, P53: tumor protein p53, ZEA: Zearalenone, Z-VAD-fmk: carbobenzoxy-valyl-alanyl-aspartyl-[O-methyl]- fluoromethylketone, Z-DEVD-fmk: Z-D(OMe)E(Ome)VD(OMe)-FMK. Z-DEVD-fluoromethylketone. Z-DEVD-fluoromethyl ketone. specific inhibitor of caspase-3, α-ZEL: alpha-zearalenol, β-ZEL: beta-zearalenol.

**Table 12:** Natural bioactive compounds dose, time of pretreatment, compound, and effect.

	Dose	Time	Compound	Effects	References
AA	6 μM	2 h pretreatment	Curcumin	Increased cell viability; Alleviated oxidative stress; GRP78, P-PERK, and P-eIF2α expression decreased; Blocked PERK-eIF2α signaling activation; Suppressed tau hyperphosphorylation; Mitigated neuronal apoptosis.	[42]
	0, 0.25, 0.5, and 1 mM	30 min pretreatment	Caffeine	Attenuated AA-inhibited phosphorylation of MAPK;	[44]
	50 µM	1 h	Z-VAD-fmk	Abolished caspase-3 activities, lowered LDH leakage, and increased cell viability	[50]
	0.123, 0.370, 1.11, 3.33, and 10.0 μg/mL	20 h	Triphala	Decreased the level of free radicals; Neuroprotective	[73]
BEA, α-ZEL and β-ZEL		24 h and 48 h	*Allium sativum* L. garlic	Cell viability increased	[60]
BEA		24 h and 48 h	Coffee by-products (coffee silverskin and spent coffee)	Cytoprotection	[58]
FB1		24 h	NAC	ROS generation at 24 h	[65]
BEA			Lutein, zeaxanthin, and goji berries extract	Cytoprotection	[62]
FB1 and OTA		24 h	Beetroot extract	Increased cell viability	[67]

AA: acrylamide, eIF2α: Eukaryotic Initiation Factor 2 alpha, GRP78: glucose regulated protein 78, LDH: lactate dehydrogenase, MAPKs: mitogen-activated protein kinases, P: Phospho, PERK: protein kinase, Pyk2: proline-rich tyrosine kinase 2 RNA (PKR)-like/Pancreatic ER Kinase, U-1240 MG: Human glioblastoma astrocytoma cells. Z-VAD-fmk: Z-Val-Ala-Asp (OMe)-FMK.

## Data Availability

Data is contained within the article.
